# Comparative Study on Nutritional Characteristics and Volatile Flavor Substances of Yak Milk in Different Regions of Gannan

**DOI:** 10.3390/foods12112172

**Published:** 2023-05-27

**Authors:** Guowu Yang, Juanxiang Zhang, Rongfeng Dai, Xiaoyong Ma, Chun Huang, Wenwen Ren, Xiaoming Ma, Jianwei Lu, Xue Zhao, Ji Renqing, Lao Zha, Xian Guo, Min Chu, Yongfu La, Pengjia Bao, Chunnian Liang

**Affiliations:** 1 Key Laboratory of Yak Breeding Engineering of Gansu Province, Lanzhou Institute of Husbandry and Pharmaceutical Sciences of Chinese Academy of Agricultural Sciences, Lanzhou 730050, China; guowu202302@163.com (G.Y.); juanxiangzhang@163.com (J.Z.); rongfengdai123@163.com (R.D.); mxy15609445561@163.com (X.M.); johnchun825@163.com (C.H.); rww15806106435@126.com (W.R.); maxiaoming@caas.cn (X.M.); guoxian@caas.cn (X.G.); chumin@caas.cn (M.C.); layongfu@caas.cn (Y.L.); baopengjia@caas.cn (P.B.); 2 Key Laboratory of Animal Genetics and Breeding on Tibetan Plateau, Ministry of Agriculture and Rural Affairs, Lanzhou 730000, China; 3 Zogaidoma Township Animal Husbandry Station of Hezuo City, Hezuo 747003, China; lujianwei0417@163.com (J.L.); zhalao2022@163.com (L.Z.); 4 Quality and Safety Inspection Center of Agricultural and Livestock Products in Hezuo, Hezuo 747099, China; zhaox0417@163.com; 5 Zogemanma Town Animal Husbandry and Veterinary Station, Hezuo 747099, China; rqingji@163.com

**Keywords:** yak milk, GC-IMS, amino acids, flavor substances

## Abstract

This study aimed to investigate the nutritional properties of yak milk in various areas of Gannan. The milk composition analyzer, automatic amino acid analyzer, and flavor analyzer were used to detect the conventional nutrients, amino acids, and volatile flavor substances of 249 yak milks in Meiren grassland, Xiahe grassland, and Maqu grassland (hereinafter referred to as Meiren yak, Xiahe yak, and Maqu yak) in the Gannan area. The results showed that the fat content of Meiren yak milk was significantly higher than that of Maqu yak and Xiahe yak (*p* < 0.05). The protein content of Meiren yak milk was significantly higher than that of Xiahe yak (*p* < 0.05), but not significantly different from that of Maqu yak (*p* > 0.05). The casein content in the milk of Maqu yak was significantly higher than that of Meiren yak and Xiahe yak (*p* < 0.05). There was no significant difference in the lactose content of yak milk in the three regions (*p* > 0.05). The content of glutamic acid in the milk of Meiren yak, Xiahe yak, and Maqu yak was noticeably high, which was 1.03 g/100 g, 1.07 g/100 g, and 1.10 g/100 g, respectively. The total amino acid (TAA) content was 4.78 g/100 g, 4.87 g/100 g, and 5.0 g/100 g, respectively. The ratios of essential amino acids (EAA) and total amino acids (TAA) in the milk of Meiren yak, Xiahe yak, and Maqu yak were 42.26%, 41.27%, and 41.39%, respectively, and the ratios of essential amino acids (EAA) and nonessential amino acids (NEAA) were 73.19%, 70.28%, and 70.61%, respectively. In the yak milk samples collected from three different regions, a total of 34 volatile flavor compounds were detected, including 10 aldehydes, five esters, six ketones, four alcohols, two acids, and seven others. The main flavor substances qualitatively obtained from Meiren yak milk were ethyl acetate, n-valeraldehyde, acetic acid, heptanal, and n-hexanal. Xiahe yak milk mainly contains ethyl acetate, isoamyl alcohol, n-valeraldehyde, heptanal, and ethyl butyrate. Maqu yak milk mainly contains ethyl acetate, n-valeraldehyde, isoamyl alcohol, heptanal, ethyl butyrate, and n-hexanal. Principal component analysis showed that the flavor difference between Xiahe yak and Maqu yak was small, while the flavor difference between Xiahe yak, Maqu yak, and Meiren yak was large. The findings of this research can serve as a foundation for the future advancement and application of yak milk.

## 1. Introduction

The yak is mainly distributed in the high-altitude area of 2500~6000 m, and can fully adapt to extremely harsh environments, such as low temperature, high altitude, and strong ultraviolet radiation [1]. Yak meat, milk, and fur are the main products and living materials of herdsmen, as well as their main economic sources. In the past, Tibetans primarily consumed yak milk, which is often referred to as “liquid gold.” This yak milk and its dairy products provided the majority of the energy, vitamins, and nutrients necessary for Tibetans [2]. Yak milk contains higher levels of dry matter, milk fat, milk protein, and other nutrients compared to regular milk. Among them, yak casein is not only a source of hypotensive peptides, but also a typical dietary protein, which can be used in a variety of high value-added functional diets [3]. Yak milk is a valuable source of nutrients, which makes it an ideal raw material for producing a variety of dairy products.

Flavor is one of the main factors to judge food. In the process of food processing, storage, and new product development, flavor is a factor that must be considered. Flavor substances are mainly perceived by human smell, including alcohols, acids, esters, aldehydes, ketones, phenols, olefins, and other volatile flavor substances [4]. The unique living environment of yaks results in a distinct flavor in their milk, setting it apart from other animals’ milk. Additionally, the flavor composition of yak milk is influenced by environmental factors such as altitude and the composition of their forage. The Meiren Prairie is a part of the distinct alpine meadow grassland landform found in the Qinghai-Tibet Plateau, with an average altitude of 3600 m. The altitude of most areas in Maqu County is 3500~3800 m, and the climate is cold. The natural grassland is dominated by alpine meadow and alpine swamp meadow (lowland meadow) [5]. The altitude of natural grassland in Xiahe County is 3000~3400 m, which is mainly divided into mountain meadow, alpine meadow, and temperate grassland [6]. According to relevant research, the higher the altitude at which yaks live, the greater the fat and protein content in their milk. As a result, there are noticeable variations in the flavor of yak milk depending on the altitude at which it is produced [7]. On this basis, this paper uses other techniques to detect amino acids and flavor substances.

Studies have shown that volatile flavor compounds are the main flavor components of milk and dairy products and are essential for the production of high-quality products. They are also a major factor in determining consumer acceptance and preference. Gas chromatography-ion migration spectrometry (GC-IMS) is a contemporary technique used for detecting flavors. This method offers several advantages, including high separation efficiency, excellent selectivity, and heightened sensitivity [8]. It is commonly employed in the detection of flavor substances found in milk and food. After all, milk and dairy products with good flavor characteristics are synonymous with quality [9]. In the last few years, the production of yak milk has grown steadily, and people are becoming more interested in its benefits. Despite this increased attention, research on yak milk remains in its early stages. This study aims to compare and analyze the main nutritional components, amino acids, and flavor substances of yak milk at different altitudes in Gannan. The findings will provide a foundation for the development and utilization of yak milk in the region.

## 2. Materials and Methods

### 2.1. Sample Collection and Processing

Yak milk samples were collected in August 2022 from Meiren Prairie (35.06° E, 103.23° N, Altitude: 3600 m), Sangke Grassland in Xiahe County (35.01° E, 102.57° N, Altitude: 3380 m), and Maqu County Pasture (34.23° E, 102.11° N, Altitude: 3680 m) in Gannan Tibetan Autonomous Prefecture, Gansu Province (in this paper, short name Gannan), respectively. All yak breeds belong to Gannan yak. The latest statistics show that there are about 30,000 yaks in the Meiren Grassland, about 90,000 yaks in the Sangke Grassland in Xiahe County, and about 560,000 yaks in the pastures of Maqu County. The feeding method of the yaks used in the experiment was pure grazing. The yak type belongs to both milk and meat type. The number of times lactation of yaks was used to collect milk samples was 2–3 times. A total of 249 (Maqu yak 102, Xiahe yak 109, Meiren yak 38) milk samples were collected and transferred into 50 mL centrifuge tubes, which were then frozen at −20 °C prior to analysis. To prepare the samples for determination and analysis, they were preheated in a water bath at 40 °C for 30 min, thawed, and gently stirred until fully homogeneous.

### 2.2. Yak Milk Composition Detection

The MilkoScanTM FT120 (Danish FUCHS Analytical Instruments Ltd., Hellerup, Denmark) was used to determine the content of conventional chemicals in yak milk, including casein, fat, lactose, protein, non-fat milk solids, and total solids. The results were then output. Each group had 3 parallel replicates.

### 2.3. Detection of Amino Acids in Yak Milk

Amino acids were determined according to GB/T 18246–2000. The Biochrom30+ automatic amino acid analyzer (Biochrom Ltd., Cambridge, UK) was used to analyze the hydrolysis products of amino acids. The column was PEEK\Na, the column temperature was 45~98 °C, the reaction was carried out at 135 °C, and the citric acid (Sinopharm Chemical Reagent Co., Ltd., Shanghai, China) buffer was delivered at a rate of 45 mL/h. Each group had 3 parallel replicates.

### 2.4. Detection of Volatile Flavor Substances in Yak Milk

The GC-IMS (FlavourSpecR, Dortmund, Germany) technique was utilized to determine the volatile flavor compounds present in yak milk. The specific operation was carried out according to the method of Li et al. [10]: a total of 2 mL of the milk sample was transferred from the sterile bottle to a 20 mL headspace bottle. The bottles were then incubated at 60 °C for 20 min. After incubation, 500 μL of sample (volume of the headspace gas) was injected into the instrument. The temperature of the syringe in the instrument was maintained at 85 °C. The FS-SE-54-CB-1 column (15 m × 0.53 mm, 1 μm) was maintained at 60 °C, and N2 (purity ≥ 99.999%) was used as the carrier gas for chromatographic separation. The carrier gas flow rate was 5 mL/min (0–2 min), 5–15 mL/min (2–10 min), 15–100 mL/min (10–20 min), and 100 mL/min (20–30 min), and the whole analysis process took a total of 30 min. Each group randomly selected samples for fusion, and each group had three parallel replicates.

### 2.5. Statistical Analysis

Excel was used to summarize the data, and then SPSS25.0 software (IBM, Armonk, NY, USA) was used to perform One-Way ANOVA analysis of variance and LSD multiple comparisons on all data. The results were expressed as mean ± standard deviation. *p* < 0.05 indicated significant difference. Compounds were identified through the analysis software LAV (Laboratory Analytical Viewer, version 2.2.1) and qualitative software GC × IMS Library Search (built-in NIST2014, IMS database) of the GC-IMS instrument. The plug-in Reporter in LAV was used to compare the GC-IMS spectra of samples, and the plug-in Gallery Plot was used to compare the GC-IMS fingerprints. Dynamic principal component analysis was performed through the Dynamic PCA plug-in that came with the instrument software.

## 3. Results

### 3.1. The Main Nutrient Composition of Yak Milk in Different Regions

The test results of the main nutritional components of the yak milk samples are shown in Table 1. In the same sampling period, there were some differences in the content of yak milk components in different regions. The fat content was 4.20–6.43%, and the fat content of Meiren yak milk was 6.43%, which was significantly higher than that of Xiahe yak and Maqu yak (*p* < 0.05). The protein content was between 4.90% and5.45%, and the protein content of Meiren yak milk was 5.45%, which was significantly higher than that of Xiahe yak (*p* < 0.05), but not significantly different from that of Maqu yak (*p* > 0.05). The lactose content is 5.06–5.18%, and there is no significant difference in yak milk in the three regions (*p* > 0.05). Yak milk was investigated in different regions of China, including Qinghai [11], Xinjiang [12], and Tibet [13]. It was reported that the contents of protein, lactose, fat, solids-not-fat, and total solids in yak milk were 4.30–5.28%, 5.01–5.21%, 4.63–6.72%, 8.27–12.11%, and 14.84–17.0% [11,12,13], respectively. This is consistent with the detection results of this experiment. In this study, there were significant differences in fat, protein, casein, non-fat milk solids, and total solids content of yak milk samples from different locations in Gannan (*p* < 0.05). Changes in the relative proportions of these milk components can be related to most factors, such as variety, diet, season, altitude, lactation stage, health status, and frequency of milking [14,15].

### 3.2. Amino Acid Composition of Yak Milk in Different Regions

Table 2 shows the detection results of 17 amino acids in yak milk samples. It can be seen from the table that the most amino acids content is consistent with the previous research results, but there are differences in a few amino acids, such as glutamic acid (1.14 g/100 g) and leucine (0.52 g/100 g) [12]. It can be seen from the test results that the glutamic acid content was the highest, followed by Meiren yak 1.03 g/100 g, Xiahe yak 1.07 g/100 g, and Maqu yak 1.10 g/100 g. The glutamic acid content in the milk of Maqu yak was significantly higher than that of Meiren yak (*p* < 0.05), and glutamic acid had important biological effects. The proportion of glutamic acid in total amino acids in yak milk from different regions was 21.55%, 21.97%, and 21.78%, respectively, with the highest in Xiahe yak. This may be related to the higher glutamic acid content of casein in yak milk than whey protein. The content of leucine in the milk of Meiren yak, Xiahe yak, and Maqu yak was 0.45 g/100 g, 0.47 g/100 g, and 0.48 g/100 g, respectively. The leucine content in the milk of Meiren yak was significantly lower than that of Xiahe yak and Maqu yak (*p* < 0.05). It is reported that the highest amino acids content in yak milk protein is glutamic acid, which is the same as bovine milk protein [16].

The total amino acids (TAA) in the milk of Meiren yak, Xiahe yak, and Maqu yak were 4.78 g/100 g, 4.87 g/100 g, and 5.05 g/100 g, respectively. There was no significant difference in TAA between Maqu yak and Xiahe yak milk (*p* > 0.05). There was no significant difference in TAA between Xiahe yak and Meiren yak milk (*p* > 0.05), while TAA in Maqu yak milk was significantly higher than that in Meiren yak milk (*p* < 0.05). The total amount of essential amino acids (EAA) in the milk of Meiren yak was 2.02 g/100 g, Xiahe yak was 2.01 g/100 g, and Maqu yak was 2.09 g/100 g, and EAA/TAA was 42.26%, 41.27%, and 41.39%, respectively. The EAA/TAA of Meiren yak milk was the highest. Among the EAA, leucine content was the highest, with 0.45 g/100 g, 0.47 g/100 g, and 0.48 g/100 g, respectively. The leucine content in the milk of Meiren yak was significantly lower than that of Xiahe yak and Maqu yak (*p* < 0.05). In addition, studies have shown that the EAA/TAA in human milk accounts for about 40%, while the EAA/TAA in yak milk in the three regions is higher than 40% in the test results [17]. Therefore, yak milk can be an excellent source of EAA for the human body. The EAA/NEAA were 73.19%, 70.28%, and 70.61%, respectively, and the highest was in Meiren yak milk.

**Figure 1:**
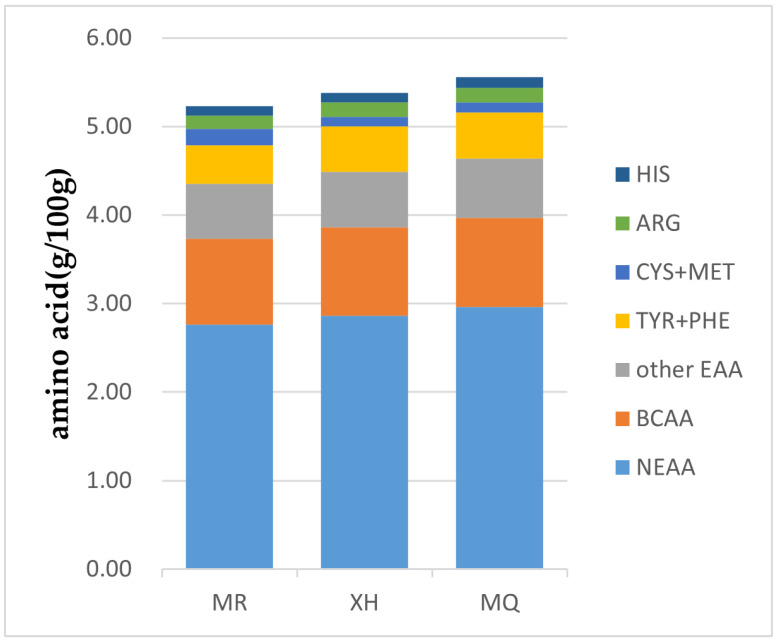
Relative contributions of individual amino acids to TAA for yak milk sampled from different regions in Gannan. HIS, histidine; ARG, arginine; CYS + MET, cystine + methionine; TYR + PHE, tyrosine + phenylalanine; Other EAA, other essential amino acids; BCAA, branched-chain amino acids; NEAA, non-essential amino acids; MR, Meiren yak; XH, Xiahe yak; MQ, Maqu yak.

Figure 1 shows the relative contribution of individual amino acids to EAA in yak milk from three regions of Gannan. From the diagram, it can be directly observed that the amino acid composition of yak milk is different between geographical regions. From the diagram, it can be seen that the CYS + MET in the milk of Meiren yak is relatively high, in which cysteine can increase glutathione levels, thus showing strong antioxidant properties and helping the body fight various diseases [18]. It can be seen from Table 2 and Figure 1 that there was no significant difference in branched chain amino acids (BCAAs) in yak milk from different regions (*p* > 0.05), but it was relatively high in Maqu yak milk. A large number of animal studies have shown that insufficient intake of BCAAs can lead to impaired immune function [19]. BCAAs play an important role in maintaining tissue growth and repair and preventing catabolism in exercise [20]. In this paper, the results are consistent with the conclusions reported in the literature of goat, Bactrian camel, sheep, and cow milk, and the amino acid with the highest content is glutamic acid [21,22]. It can be seen from Table 2 that there was no significant difference in histidine and arginine in amino acids of yak milk in different regions of Gannan (*p* > 0.05), but arginine was higher in Maqu yak milk. Histidine is an important part of protein synthesis in the body, and it is also involved in some important biological metabolic processes, so it is very important for human growth and health maintenance [23]. Arginine is necessary for premature infants, and has a significant influence on the growth and health of premature infants. It is involved in a variety of metabolic pathways in the human body, including fatty acid metabolism, cholesterol synthesis, and nitric oxide production [24]. Arginine deficiency in premature infants can lead to hyperammonemia and cardiovascular, pulmonary, and neurological dysfunction [25].

### 3.3. Composition of Volatile Substances in Yak Milk from Different Regions

**Figure 2:**
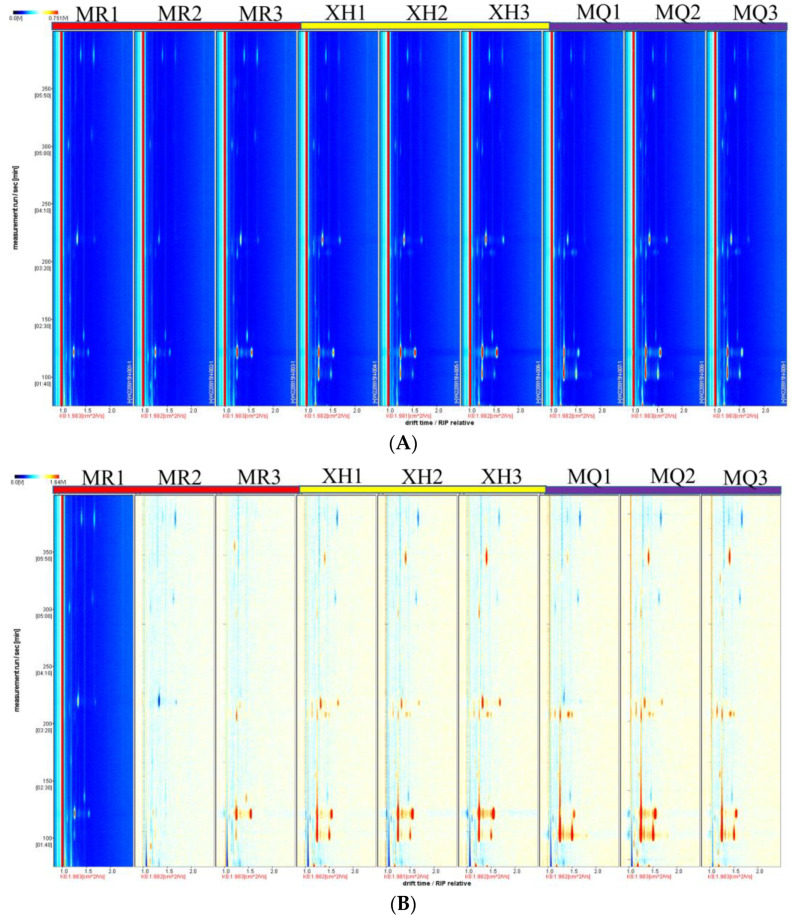
(**A**) Ion migration spectra of yak milk in three regions of Gannan; (**B**) Difference map; MR, Meiren yak; XH, Xiahe yak; MQ, Maqu yak.

The volatile flavor compounds of yak milk samples from three regions were analyzed by GC-IMS. The ion migration spectra of volatile components in yak milk from three regions were obtained by normalizing the ion migration time and the position of the reaction ion peak (RIP) (Figure 2A). Each point in the figure represents a specific volatile compound. The signal intensity of each volatile compound represents the concentration level. The deeper the red, the higher the concentration, and the white is the opposite. As shown in the figure, the volatile compounds in three different raw milks were well separated by GC-IMS. It can be seen from the comparison difference map that there are differences in the volatile flavor composition of yak milk in different regions. The concentration and types of volatile compounds in Xiahe yak milk and Maqu yak milk are similar, but are quite different from Meiren yak milk (Figure 2B).

#### 3.3.1. Qualitative Analysis and Fingerprint of Volatile Substances

**Figure 3:**
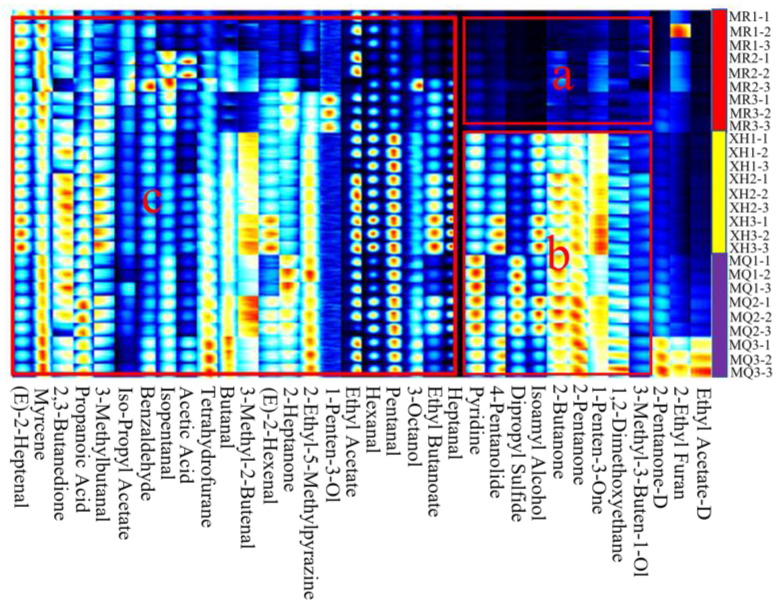
Fingerprints of volatile substances in gas phase ion mobility spectra of yak milk in three regions (**a**–**c**). Note: Rows represent samples. Columns represent volatile species. There is a total of three repetitions per set. The darker the spot, the greater the content of volatile species (“D” for dimer). MR, Meiren yak; XH, Xiahe yak; MQ, Maqu yak.

According to the drift time and retention time of volatile substances, external standard ketones C4–C9 (2-butanone, 2-pentanone, 2-hexanone, 2-heptanone, 2-octanone, 2-nonanone) were used to refer to. A total of 34 volatile flavor compounds were detected in yak milk from the three regions, including 10 aldehydes, five esters, six ketones, four alcohols, two acids, and seven others (Table 3). Due to differences in compound concentrations in milk samples, a small number of single compounds may generate two or multiple signals (dimers or trimers) [26]. To better compare the differences in the content of volatile compounds in yak milk in the three regions, we selected the peaks in the GC-IMS two-dimensional spectrum to automatically generate fingerprints (Figure 3). To recognize the characteristic peak areas of different yak milk, the rows in the figure represent the detected substances, and the columns represent the content of the same volatile substance in different milk samples. Each bright spot represents a flavor substance, and the depth of the color represents the content of the flavor substance. The brighter the color, the higher the content. As shown in Figure 3, compared with region B, the content of volatile substances such as pyridine, γ-valeryl, dipropyl sulfide, and isoamyl alcohol in the milk of Meiren yak was significantly lower than that of Xiahe yak and Maqu yak in region A. The content of most substances in region C was less different in yak milk from the three regions.

Aldehydes, esters, ketones, and alcohols are important volatile flavor substances in yak milk. The aldehydes detected mainly include pentanal, heptanal, hexanal, isopentanal, butyraldehyde, and benzaldehyde. Quantitative results were analyzed by Figure 3 and Table 3. Aldehydes mainly come from the oxidation reaction of milk fat. Because of their low flavor threshold, the flavor characteristics are obvious [27,28]. Relevant studies have shown that aldehydes and ketones are generally used as indicators for evaluating the oxidative flavor of milk and dairy products [29]. Hexanal presents fruity and grassy flavors [30]. Heptaldehyde has a special milky and oily flavor [31]. The relative contents of hexanal and heptanal in the flavor substances of Xiahe yak milk were significantly higher than those of Meiren yak and Maqu yak (*p* < 0.05). Benzaldehyde may be produced by the Streckel amino acid reaction of amino acids and has been identified as the main monocarbonyl compound of roasted peanuts. It has pleasant almond, nut, and fruit aromas [32], and plays an important role in the formation of the overall good flavor of milk, and there is no significant difference in yak milk in the three regions (*p* > 0.05).

The detected ketones mainly included 2-butanone, 2-pentanone, 2-heptanone, 2,3-butanedione, and 1-penten-3-one. The relative content of ketones in Maqu yak milk was higher. Among them, 2-butanone and 2-pentanone have fruity, sweet, and slight milk aromas [33], both of which are relatively high in Maqu yak milk. 2,3-butanedione has a very abundant cream flavor [34]. The substance produced by linoleic acid through oxidation reaction is mainly 2-heptanone [35], giving yak milk flavor and sweet flavor. It is a representative volatile flavor substance of yak milk flavor [36]. The relative content of 2,3-butanedione in Meiren yak milk was significantly lower than that in Xiahe yak and Maqu yak (*p* < 0.05). The relative content of 2-heptanone in the milk of Maqu yak was significantly higher than that of Meiren yak and Xiahe yak (*p* < 0.05). Therefore, it shows that the milk flavor in Maqu yak milk is rich.

The detected esters mainly included ethyl acetate, ethyl butyrate, isopropyl acetate, and γ-valeryl. The relative content of esters in Meiren yak milk was higher. The relative content of ethyl acetate in the flavor substances of different yak milks was the highest, and the difference of ethyl acetate in the flavor substances of different yak milks in the three regions was not significant (*p* > 0.05). The relative content of ethyl butyrate in Xiahe yak milk was significantly higher than that in Meiren yak and Maqu yak (*p* < 0.05). Ethyl acetate and ethyl butyrate have sweet fruit flavors [33], and lactones have creamy and oily flavors [37]. Therefore, esters give yak milk a rich, fruity aroma.

The alcohols detected mainly included isoamyl alcohol, 1-penten-3-ol, 3-octanol, and 3-methyl-3-buten-1-ol. Isoamyl alcohol provides mellow, banana, and other flavors for yak milk [38]. 1-penten-3-ol has a cooked meat flavor [39]. Acids, including acetic acid and propionic acid, mainly present the smell of cheese. In addition, dipropyl sulfide, pyridine, ethylene glycol dimethyl ether, myrcene, 2-ethylfuran, tetrahydrofuran, and 2-ethyl-5-methylpyrazine were also detected in the samples. 2-ethylfuran has coffee and nut aromas. The taste threshold of pyrazine compounds is low, with nutty and toasty flavors [10].

#### 3.3.2. PCA Results of Yak Milk GC-IMS in Different Regions

**Figure 4:**
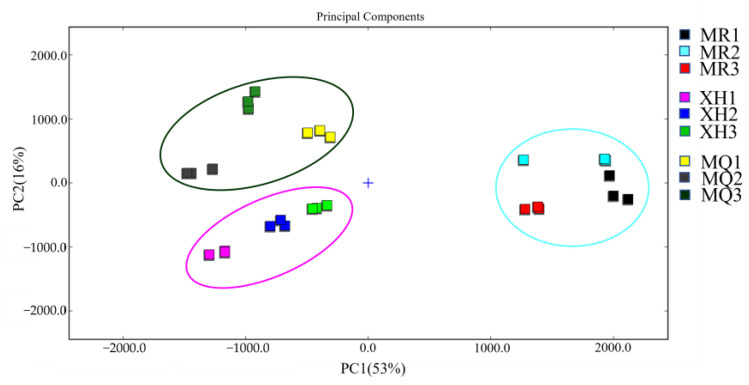
PCA of volatile compounds in yak milk from different regions. MR, Meiren yak (Blue circle); XH, Xiahe yak (Purple circle); MQ, Maqu yak (Green circle).

Through the Dynamic PCA processing of the instrument software, the yak milk in different regions can be distinguished more intuitively. As shown in Figure 4, the contribution rates of PC1 and PC2 are 53% and 16%, respectively, and the total of PC1 and PC2 is 69%. The results of SPSS software are similar, indicating that the results are effective. It can be seen from the figure that the distance between Xiahe yak and Maqu yak is close, indicating that there is little difference in flavor between them. The distance from Meiren yak is far, indicating that there is a big difference in flavor between Xiahe yak and Maqu yak.

## 4. Discussion

The content of nutrients in yak milk varies with grazing conditions, regional altitude, seasonal changes, and grassland types. There are some differences in grassland types, forage quality, and seasonal changes in different altitude areas, so that the nutrients of yak milk will also be different [7]. In this paper, yaks in three different regions of Gannan were taken as the research object, and the differences in nutritional components and volatile flavor substances in yak milk in different regions and pastoral areas of Gannan were analyzed. The results showed that the average altitude of Meiren grassland and Maqu County was higher, and the content of fat and protein in yak milk was the highest. Xiahe County has the lowest altitude and the lowest fat and protein content of yak milk. Therefore, the altitude of the yak area has a certain influence on its milk fat and protein content [7].

Milk fat is a high-quality lipid substance in milk and contains a lot of fat-soluble vitamins. The digestibility of milk fat in the human gastrointestinal tract can reach more than 98% [40], which is an important index to measure milk quality. Similarly, the intake of protein and fat plays a very important role in the growth and development of mammals. High protein levels can provide essential amino acids for the growth of newborns, and some special proteins can also improve immunity and promote the utilization of trace elements [41]. In this study, the fat and protein content of Meiren yak milk reached 6.43% and 5.45%. Therefore, Meiren yak milk is more suitable for newborns. Lactose can be decomposed into glucose and galactose by small intestinal lactase. Glucose and galactose are both monosaccharides that can be directly absorbed by the human body [42,43]. Milk protein mainly includes casein and whey protein [44]. The casein content of Maqu yak milk is 4.40%, accounting for 84.6% of the total protein. Casein is a phosphorus-containing acidic protein synthesized by the mammary gland itself. It is the main nutritional protein in milk and a rich source of calcium and phosphorus in milk [45].

Amino acids are the basic unit of protein and a class of small molecule metabolites, so amino acids also become a bridge between proteins and metabolites [46]. The amino acids in milk not only constitute proteins, but also play a crucial role in metabolism and growth and development, such as regulating the activities of organisms, exerting immune activity, and synthesizing various bioactive substances through metabolism [47]. The results showed that among the non-essential amino acids, the content of glutamic acid in the milk of Meiren yak, Xiahe yak, and Maqu yak was the highest, which was 1.03 g/100 g, 1.07 g/100 g, and 1.10 g/100 g, respectively, and was consistent with the results of Hu et al. [2]. Studies have found that glutamic acid is a flavor amino acid [48]. Glutamate and glutamine are sources of α-ketoglutarate in the citric acid cycle and the main components of neurotransmitters in the brain [49]. Garattini [50] found that glutamate, as an important neurotransmitter, also plays an important role in carbohydrate and fat metabolism. Among the essential amino acids, the content of leucine in the milk of Meiren yak, Xiahe yak, and Maqu yak was the highest, which was 0.45 g/100 g, 0.47 g/100 g, and 0.48 g/100 g, respectively. Leucine and valine in essential amino acids are two kinds of BCAAs, which usually account for 15–25% of protein intake. Dairy products are rich sources of branched-chain amino acids [51]. Lysine is the first essential amino acid in the human body and plays an important role in human metabolism [52,53]. The catabolism of lysine is usually carried out in the liver, and the yeast amino acid is formed by condensation with ketoglutarate; the formed yeast amino acid is converted into L-α-aminoadipate semialdehyde, and the final product is acetyl-CoA [54]. Lysine differs from other amino acids. Lysine does not participate in transamination, and the deamination reaction of lysine is irreversible, so lysine is a very special class of amino acids in catabolism [55]. Lysine is an amino acid that produces sugar and ketones, so it can participate in the formation of D-glucose, lipids, and other substances, and finally, produce energy. In this study, the relative content of lysine in the milk of Meiren yak, Xiahe yak, and Maqu yak was 8.58%, 8.62%, and 8.91%, respectively, and the relative content of lysine in Maqu yak milk was the highest.

The EAA/TAA in the milk of Meiren yak, Xiahe yak, and Maqu yak was 42.26%, 41.27%, and 41.39%, respectively, and the EAA/NEAA was 73.19%, 70.28%, and 70.61%, respectively, which was close to the EAA content (40%) and E/N value (0.6) specified by the FAO/WHO standard [56], indicating that the milk of yak in these three regions belongs to high-quality protein resources. At the same time, the nutritional level of amino acids in yak milk is not entirely dependent on the content of amino acids, but also on the proportion of EAA [57]. The EAA/TAA% ratio of Meiren yak milk was the highest.

The flavor of yak milk is affected by many factors, such as physiological factors, altitude, forage composition, and storage. For example, the physical and chemical properties of Tianzhu white yak milk and Gannan yak milk were studied. The results showed that lactation parity and lactation time had a great influence on the nutritional composition and sensory quality of yak milk [58]. Ketones have obvious flavor characteristics and are typical volatile flavor substances in many varieties of milk. Among them, 2-heptanone has a milky and sweet flavor, which is a representative volatile flavor substance of milk flavor [31]. Aldehydes are the products of a series of reactions of milk fat oxidation. Due to the low flavor threshold and the flavor of milk and oil, they are also common characteristic components of milk flavor [59]. (E)-2-heptenal is also a common volatile component in yak milk, which mainly has fat flavor [60]. The frankincense flavor of yak milk is generally stronger than that of ordinary milk, mainly because the fat content of yak milk is more abundant. Fat composition and content are important factors affecting the sensory quality of yak milk. The formation of milk flavor substances is mainly produced by the degradation of fat and protein. Esters in flavor substances usually have fruity and sweet aromas. These compounds are typically formed through the esterification reaction between milk fatty acids and alcohols [61]. Esters are common volatile flavor substances in yak milk and its dairy products and are also the main source of its unique flavor.

## 5. Conclusions

This study compared the nutritional characteristics and volatile flavor substances of yak milk in three different regions of Gannan. The results showed that Meiren yak milk had higher fat and protein contents. There was no significant difference in the lactose composition of yak milk in the three regions (*p* > 0.05). The TAA in the milk of Meiren yak, Xiahe yak, and Maqu yak were 4.78 g/100 g, 4.87 g/100 g, and 5.05 g/100 g, respectively. A total of 34 volatile flavor compounds were identified in yak milk from the three regions, including aldehydes, esters, ketones, alcohols, acids, and others. By drawing GC-IMS fingerprints, the characteristic peak areas of yak milk in different regions were clearly analyzed. Through PCA processing, it was found that there was little difference in flavor substances between Xiahe yak and Maqu yak, but both of them were significantly different from Meiren yak.

## Figures and Tables

**Table 1:** Chemical composition of yak milk from three different regions of Gannan.

Item (%)	Region ^1^		
	MR	XH	MQ
Casein	3.97 ± 0.15 ^b^	4.06 ± 0.17 ^b^	4.40 ± 0.10 ^a^
Fat	6.43 ± 0.32 ^a^	4.20 ± 0.15 ^c^	5.22 ± 0.40 ^b^
Lactose	5.06 ± 0.05 ^a^	5.18 ± 0.04 ^a^	5.13 ± 0.07 ^a^
Protein	5.45 ± 0.12 ^a^	4.90 ± 0.23 ^b,c^	5.20 ± 0.21 ^a,b^
SNF ^2^	9.57 ± 0.18 ^b^	11.50 ± 0.27 ^a^	11.63 ± 0.23 ^a^
Total Solids	13.97 ± 0.36 ^c^	15.45 ± 0.39 ^b^	18.57 ± 0.39 ^a^

^1 a,b,c^ Data with different superscript letters in the same row indicate a significant difference (*p* < 0.05). MR, Meiren yak, *n* = 38; XH, Xiahe yak, *n* = 109; MQ, Maqu yak, *n* = 102; ^2^ Solids-not-fat. Data are presented as the mean ± standard deviation.

**Table 2:** Amino acid compositions in yak milk from three different regions of Gannan.

AA ^2^ (g/100 g)	Region ^1^		
	MR	XH	MQ
Histidine	0.11 ± 0.006 ^a^	0.11 ± 0.006 ^a^	0.12 ± 0.000 ^a^
Isoleucine	0.22 ± 0.006 ^a^	0.23 ± 0.012 ^a^	0.23 ± 0.006 ^a^
Leucine	0.45 ± 0.023 ^b^	0.47 ± 0.010 ^a^	0.48 ± 0.006 ^a^
Valine	0.30 ± 0.010 ^a^	0.30 ± 0.006 ^a^	0.30 ± 0.017 ^a^
Methionine	0.09 ± 0.025 ^a^	0.03 ± 0.006 ^b^	0.04 ± 0.012 ^b^
Threonine	0.21 ± 0.006 ^a^	0.21 ± 0.006 ^a^	0.22 ± 0.005 ^a^
Phenylalanine	0.22 ± 0.023 ^b^	0.24 ± 0.021 ^a^	0.25 ± 0.012 ^a^
Lysine	0.41 ± 0.010 ^b^	0.42 ± 0.012 ^b^	0.45 ± 0.012 ^a^
Total EAA	2.02 ± 0.019 ^a^	2.01 ± 0.035 ^a^	2.09 ± 0.021 ^a^
Serine	0.28 ± 0.010 ^b^	0.29 ± 0.006 ^a,b^	0.30 ± 0.010 ^a^
Glutamic acid	1.03 ± 0.026 ^b^	1.07 ± 0.020 ^a,b^	1.10 ± 0.025 ^a^
Glycine	0.08 ± 0.001 ^a^	0.08 ± 0.000 ^a^	0.08 ± 0.003 ^a^
Alanine	0.13 ± 0.002 ^a^	0.14 ± 0.001 ^a^	0.14 ± 0.006 ^a^
Cystine	0.09 ± 0.015 ^a^	0.08 ± 0.006 ^a,b^	0.07 ± 0.012 ^b^
Aspartic acid	0.36 ± 0.006 ^b^	0.35 ± 0.012 ^b^	0.38 ± 0.006 ^a^
Tyrosine	0.22 ± 0.021 ^b^	0.27 ± 0.015 ^a^	0.27 ± 0.012 ^a^
Arginine	0.15 ± 0.019 ^a^	0.16 ± 0.010 ^a^	0.17 ± 0.007 ^a^
Proline	0.42 ± 0.053 ^b^	0.42 ± 0.041 ^b^	0.45 ± 0.021 ^a^
Total NEAA	2.76 ± 0.042 ^b^	2.86 ± 0.027 ^a,b^	2.96 ± 0.049 ^a^
TAA	4.78 ± 0.057 ^b^	4.87 ± 0.050 ^a,b^	5.05 ± 0.069 ^a^
BCAAs	0.97 ± 0.036 ^a^	0.99 ± 0.015 ^a^	1.01 ± 0.026 ^a^
EAA/TAA (%)	42.26	41.27	41.39
EAA/NEAA (%)	73.19	70.28	70.61

^1 a,b^ Data with different superscript letters in the same row indicate a significant difference (*p* < 0.05). MR, Meiren yak, *n* = 38; XH, Xiahe yak, *n* = 109; MQ, Maqu yak, *n* = 102; ^2^ EAA, essential amino acid; NEAA, non-essential amino acid; TAA, total amino acid; BCAAs, branched-chain amino acids. Data are presented as the mean ± standard deviation.

**Table 3:** Relative abundance of volatile compounds in yak milk from three regions of Gannan.

Compounds	RI ^2^	Rt ^3^ [s]	Dt ^4^	Region ^1^		
				MR	XH	MQ
Ketones (6)						
2-Butanone	596.5	50.505	1.24595	0.052 ± 0.007 ^b^	0.181 ± 0.007 ^a^	0.187 ± 0.007 ^a^
2-Pentanone	672.7	66.271	1.12129	0.112 ± 0.013 ^b^	0.110 ± 0.003 ^b^	0.334 ± 0.061 ^a^
2-Heptanone	880.4	205.561	1.25979	0.070 ± 0.012 ^b^	0.092 ± 0.002 ^b^	0.123 ± 0.006 ^a^
2,3-Butanedione	576.2	46.978	1.18614	0.101 ± 0.007 ^b^	0.129 ± 0.004 ^a^	0.121 ± 0.008 ^a^
1-Penten-3-One	611.7	53.306	1.15948	0.046 ± 0.002 ^c^	0.079 ± 0.005 ^a^	0.067 ± 0.003 ^b^
2-Pentanone dimer	644.1	59.84	1.11984	0.034 ± 0.003 ^b^	0.176 ± 0.007 ^a^	0.186 ± 0.004 ^a^
Esters (5)						
Ethyl Acetate	613.3	53.617	1.33819	4.123 ± 0.556 ^a^	5.007 ± 0.409 ^a^	4.650 ± 0.405 ^a^
Ethyl Butanoate	780.5	120.019	1.56098	0.367 ± 0.082 ^b^	0.863 ± 0.090 ^a^	0.539 ± 0.078 ^b^
Iso-Propyl Acetate	647.5	60.566	1.17533	0.743 ± 0.125 ^a^	0.384 ± 0.014 ^b^	0.294 ± 0.041 ^b^
Ethyl Acetate dimer	599.6	51.061	1.09698	0.020 ± 0.000 ^b^	0.033 ± 0.005 ^b^	0.101 ± 0.020 ^a^
4-Pentanolide	982.2	343.744	1.41125	0.038 ± 0.002 ^b^	0.147 ± 0.019 ^a^	0.158 ± 0.022 ^a^
Aldehydes (10)						
Hexanal	784.2	122.651	1.25803	0.412 ± 0.044 ^b^	0.756 ± 0.032 ^a^	0.526 ± 0.051 ^b^
Butanal	598.9	52.371	1.29905	0.103 ± 0.006 ^c^	0.170 ± 0.003 ^b^	0.207 ± 0.012 ^a^
Pentanal	693.3	72.079	1.4225	0.665 ± 0.025 ^c^	1.696 ± 0.011 ^a^	1.619 ± 0.019 ^b^
Isopentanal	691.1	71.146	1.18686	0.312 ± 0.045 ^a^	0.213 ± 0.004 ^b^	0.299 ± 0.004 ^a^
Heptanal	892.8	219.599	1.25803	0.411 ± 0.092 ^b^	0.966 ± 0.102 ^a^	0.590 ± 0.084 ^b^
Benzaldehyde	956.1	301.631	1.14956	0.121 ± 0.013 ^a^	0.119 ± 0.003 ^a^	0.114 ± 0.010 ^a^
(E)-2-Hexenal	843.2	168.294	1.17956	0.041 ± 0.004 ^c^	0.060 ± 0.005 ^a^	0.053 ± 0.004 ^a,b^
(E)-2-Heptenal	952.6	296.32	1.25246	0.138 ± 0.011 ^b^	0.162 ± 0.007 ^a^	0.111 ± 0.004 ^c^
3-Methyl-2-Butenal	780.4	119.921	1.35214	0.048 ± 0.006 ^a^	0.053 ± 0.003 ^a^	0.054 ± 0.004 ^a^
3-Methylbutanal	652.2	61.602	1.39578	0.071 ± 0.006 ^b^	0.110 ± 0.008 ^a^	0.090 ± 0.006 ^b^
Alcohols (4)						
Isoamyl Alcohol	754.1	102.868	1.51065	0.101 ± 0.015 ^b^	1.739 ± 0.122 ^a^	1.432 ± 0.175 ^a^
3-Octanol	1001.1	378.22	1.40231	0.093 ± 0.023 ^b^	0.120 ± 0.000 ^a,b^	0.157 ± 0.009 ^a^
1-Penten-3-Ol	683.6	68.894	1.36515	0.051 ± 0.009 ^a^	0.031 ± 0.001 ^b^	0.037 ± 0.002 ^a,b^
3-Methyl-3-Buten-1-Ol	724	86.236	1.18474	0.302 ± 0.119 ^a^	0.114 ± 0.006 ^a^	0.140 ± 0.009 ^a^
Acids (2)						
Acetic Acid	611.7	53.306	1.15948	0.517 ± 0.107 ^a^	0.376 ± 0.041 ^b^	0.413 ± 0.055 ^a,b^
Propanoic Acid	692.8	71.853	1.26579	0.134 ± 0.004 ^c^	0.181 ± 0.003 ^b^	0.199 ± 0.009 ^a^
Others (7)						
Pyridine	753.5	102.472	1.25281	0.161 ± 0.014 ^c^	0.657 ± 0.016 ^b^	0.948 ± 0.025 ^a^
Dipropyl Sulfide	884.6	210.185	1.1502	0.041 ± 0.003 ^c^	0.191 ± 0.011 ^b^	0.438 ± 0.034 ^a^
1,2-Dimethoxyethane	635	57.933	1.31767	0.042 ± 0.005 ^b^	0.150 ± 0.007 ^a^	0.162 ± 0.010 ^a^
2-Ethyl Furan	707.7	78.414	1.06197	0.114 ± 0.019 ^a,b^	0.081 ± 0.001 ^b^	0.126 ± 0.014 ^a^
Tetrahydrofuran	629.1	56.728	1.23082	0.211 ± 0.028 ^b^	0.248 ± 0.008 ^b^	0.341 ± 0.013 ^a^
Myrcene	988.4	354.555	1.22026	0.810 ± 0.014 ^a^	0.614 ± 0.012 ^c^	0.746 ± 0.014 ^b^
2-Ethyl-5-Methylpyrazine	1003.9	387.315	1.67288	0.147 ± 0.017 ^c^	0.310 ± 0.005 ^b^	0.347 ± 0.009 ^a^

^1 a,b,c^ Data with different superscript letters in the same row indicate a significant difference (*p* < 0.05). MR, Meiren yak, *n* = 38; XH, Xiahe yak, *n* = 109; MQ, Maqu yak, *n* = 102; ^2^ Retention index calculated using n-ketones C4–C9 as the external standard on an FS-SE-54-CB column; ^3^ Retention time in the capillary GC column; ^4^ The drift time in the drift tube. Data are presented as the mean ± standard deviation.

## Data Availability

The data presented in this study are available on request from the corresponding author.
